# In vitro validation of Digital Image Analysis Sequence (DIAS) for the assessment of the marginal fit of cement-retained implant-supported experimental crowns

**DOI:** 10.1186/s40729-021-00290-6

**Published:** 2021-02-15

**Authors:** Aristeidis A. Villias, Stefanos G. Kourtis, Hercules C. Karkazis, Gregory L. Polyzois

**Affiliations:** 1 Department of Prosthodontics, School of Dentistry, National and Kapodistrian University of Athens, Athens, Greece; 2 57-59 Kolokotroni Str., Piraeus, Greece

**Keywords:** Validation study, Observation methods, Dental marginal adaptation, Marginal fit, Cementation, Implant-supported crowns

## Abstract

**Background:**

The replica technique with its modifications (negative replica) has been used for the assessment of marginal fit (MF). However, identification of the boundaries between prosthesis, cement, and abutment is challenging. The recently developed Digital Image Analysis Sequence (DIAS) addresses this limitation. Although DIAS is applicable, its reliability has not yet been proven.

The purpose of this study was to verify the DIAS as an acceptable method for the quantitative assessment of MF at cemented crowns, by conducting statistical tests of agreement between different examiners.

**Methods:**

One hundred fifty-one implant-supported experimental crowns were cemented. Equal negative replicas were produced from the assemblies. Each replica was sectioned in six parts, which were photographed under an optical microscope. From the 906 standardized digital photomicrographs (0.65 μm/pixel), 130 were randomly selected for analysis. DIAS included tracing the profile of the crown and the abutment and marking the margin definition points before cementation. Next, the traced and marked outlines were superimposed on each digital image, highlighting the components’ boundaries and enabling MF measurements.

One researcher ran the analysis twice and three others once, independently. Five groups of 130 measurements were formed. Intra- and interobserver reliability was evaluated with intraclass correlation coefficient (ICC). Agreement was estimated with the standard error of measurement (SEM), the smallest detectable change at the 95% confidence level (SDC_95%_), and the Bland and Altman method of limits of agreement (LoA).

**Results:**

Measured MF ranged between 22.83 and 286.58 pixels. Both the intra- and interobserver reliability were excellent, ICC = 1 at 95% confidence level. The intra- and interobserver SEM and SDC_95%_ were less than 1 and 3 pixels, respectively. The Bland–Altman analysis presented graphically high level of agreement between the mean measurement of the first observer and each of the three other observers’ measurements. Differences between observers were normally distributed. In all three cases, the mean difference was less than 1 pixel and within ± 3 pixels LoA laid at least 95% of differences. *T* tests of the differences did not reveal any fixed bias (*P* > .05, not significant).

**Conclusion:**

The DIAS is an objective and reliable method able to detect and quantify MF at ranges observed in clinical practice.

## Background

In fixed prosthodontics, it is critical that marginal fit (MF) is kept to a minimum, for successful long-term survival of the prosthesis [1–3]. Poor MF on the contrary, might accelerate cement dissolution and plaque retention, therefore increasing the risks of biological complications [4–7]. Additionally, it drastically increases the probability of luting material exposure at the restoration margin [8, 9].

Clinically accepted MF ranges from as low as 26 μm and might exceed 200 μm [10–16]. Although discrepancies at the margin, ideally, are ought to be kept to a minimum, perfect margins and absolute passive framework fit are unlikely to be achieved [17, 18].

Cementation is associated with higher risks for the health of peri-implant tissues [19–22]. The cause of this might relate to the fact that the connective tissue barrier surrounding implant-supported prostheses has weak mechanical resistance in comparison to the natural teeth [23–25]. However, there seems to be a lack of concrete evidence for the exclusion from use of either retention type. Therefore, selection of screw or cement retention is a subject of the patient’s and the dentist’s preference [26, 27]. There are situations that cement retention is a preferable treatment choice for implant-supported crowns, despite its disadvantages. Cement retention might be selected due to esthetics, passivity of fit, occlusal management, unfavorable implant inclination, less cost, and familiarity with the procedures [28–30]. A plethora of methods have been used for the assessment of the fit of prostheses on abutments [14, 31–34]. One of the most widely used methods is the replica technique (RT), and according to which, low viscosity light-body silicone material is applied in the crown, which is then seated on the abutment simulating the cementation procedure [14]. Higher-viscosity silicone is then poured in the crown to stabilize the first thin silicone layer. After setting, the silicone replica can be sectioned and measured [33]. This straightforward and inexpensive method has been proven a reliable and accurate alternative to the destructive cross-sectional method [35, 36]. RT has also been modified by making an external impression of the fixed crown on the corresponding abutment and pouring epoxy resin material after setting of the silicone. The marginal fit was then measured on the resin replica [37].

However, the replica technique has limitations [38–40]. Manual sectioning process of the fragile replica is associated with errors such as inclined cutting, discontinuous cutting, and distortions of the layer. Such errors could give misleading results [2]. Although, cross-sectional images obtained from the slices allow assessment of the prosthesis’ marginal fit, the accuracy of the method depends on the ability of the observer to identify the boundaries between the prosthesis, cement, and abutment as well as to identify the misfit using these boundaries [40]. Furthermore, margins may appear rounded under magnification obstructing an accurate definition of the margin [33]. Also, tearing of the elastomeric film upon removal from the crown has been reported [33, 38]. Measurements of the marginal fit have also been conducted directly on the impression silicone, which functioned as a negative replica (NR) [41, 42]. The NR technique successfully addressed inherent limitations of the RT [38, 39]. Due to study specific equipment, the homogeneity of the replicas was improved, replica slicing procedure was standardized, and sharp digital microphotographs were acquired in a standardized manner. After implementing the digital image analysis sequence (DIAS), a recently developed stepwise procedure, crown margin, abutment finishing line, and the profile of the set cement were recognizable on the two-dimensional image of the replica cross section [41, 42].

The RT has been evaluated in several studies. Despite its limitations, it has been proven for its applicability, repeatability, and validity [2, 43–46]. However, measurements are often conducted by one examiner [31, 44, 45, 47]. Measurement of agreement is a prerequisite for the acceptance of a new method or process before its application. The agreement between measurements performed by different examiners, reproducibility, is described by an interobserver variation. On the contrary, the closeness between measurements by the same examiner, repeatability, is described by an intraobserver variation [48]. A method with a high degree of reproducibility and repeatability is considered precise and acceptable. A variety of statistical methods has been used to verify agreement, including Pearson’s correlation coefficient, intraclass correlation coefficient (ICC), and non-parametric tests such as Kendall’s tau and Spearman’s rho [48–53].

Agreement can also be evaluated with the standard error of measurement (SEM), the smallest detectable change at the 95% confidence level (SDC_95%_). Furthermore, the Bland and Altman method is a useful tool for the quantification of agreement between examiners by means of graphic representation of the limits of agreement at the 95% confidence level (LoA_95%_) [52, 54–58]. A reasonably large sample size is necessary for statistical tests with an adequate level of power, given the effect size, so that conclusions can be safely drawn when a method is examined [54, 59–62]. The DIAS is a recently developed and useful method for analyzing MF, while addressing some of the inherent problems encountered with the RT before. Although DIAS is applicable, its validity has not yet been proven. The purpose of this study was to verify the DIAS as an acceptable method for the evaluation of MF by conducting statistical tests of agreement between different examiners using the Bland and Altman method. The hypothesis was that the measurements with DIAS would not be different between and within different examiners.

## Methods

### Samples

A two-component abutment (CSam) (Friadent CeraBase D5.5mm, conical, Dentsply Friadent, Mannheim, Germany) was utilized as an experimental crown model. Also, a simplified sample (NSam) was designed and produced by a certified implant-component manufacturer (Novadental ltd, Athens, Greece) to reduce costs. Both samples had two parts that presented axial symmetry. One part simulated the abutment and the other the implant-supported crown. The MF was measured for both samples before cementation, according to Holmes’ definition of the “absolute marginal discrepancy”: CSam MF: 65 ± 5 μm, NSam MF: 4.5 ± 2 μm [3].

Aluminum washers of known thickness 85 ± 10 μm and 125 ± 10 μm as well as 75 ± 5-μm-thick stainless steel washers (Washers Ø4.40/3.10-mm material 1.4310, Lastec AG, Bruegg, Switzerland) were manufactured. Each washers’ thickness was verified with a micrometer caliper (0–25-mm micrometer, VIS, Poland). By incorporating these washers on the abutment, experimental crowns of known initial MF levels were created.

### Cementation

A pilot run was executed for the proof of concept of the modified negative replica technique, which also incorporated a prototype experimental equipment, as well as familiarization with the procedures and calculation of an adequate sample size for a larger experiment. In this initial run, 31 CSam were cemented with a temporary cement (TempBond NE, Kerr Italia, Scafati, Italy). One hundred twenty NSAm were cemented with permanent cements as part of a larger experiment so that the same number of specimens were included in every group: Either zinc phosphate cement (ZnP) (Hoffmann’s Cement quick setting-zinc phosphate cement, Hoffmann Dental Manufaktur GmbH, Berlin, Germany) or self-adhesive dual-curing luting composite (LCo) (Bifix SE, Voco GmbH, Cuxhaven, Germany) was used.

The cementation process took place in a customized axial loading device (ALD) (Hercules Parts Co., Athens, Greece). The ALD facilitated standardized cementation of samples. After the CSam was thoroughly cleaned, the screw orifice of the ceramic sleeve was blocked with dental wax. The titanium core was assembled on the implant analog and placed in the sample holder of the ALD. Samples were cemented with the temporary cement according to the instructions of the manufacturer. Using an insulin syringe, a standardized volume (0.014 ± 0.007 ml) of mixed cement was applied in the ceramic sleeve. The ceramic sleeve was seated on the titanium core with finger force of 5 ± 1 N applied for 5 s. Then, a constant load of 30 ± 0.2 N was applied on the crown in the ALD for at least 10 min, until the cement was completely set. Excess cement was removed either before setting with a cotton stick after sitting the crown with finger pressure or after setting with a plastic scaler. Excess cement removal was carried out within a preset time frame of 45 s. The cementation procedure was conducted by the same researcher throughout this experiment; optimal environmental conditions were kept constant (23 ± 1 °C, 50 ± 10% RH) and powder-free nitrile gloves were used.

In this way, 31 CSam were created: 11 samples of initial MF and excess cement removed after the setting with a plastic scaler, 3 samples of MF increased by 85 μm and excess cement removed after the setting with a plastic scaler, 6 samples of MF increased by 125 μm and excess cement removed after the setting with a plastic scaler, 5 samples of initial MF and excess cement removed before the setting with a cotton pellet, and 6 samples of MF increased by 85 μm and excess cement removed before the setting with a cotton pellet.

**Fig. 1:**
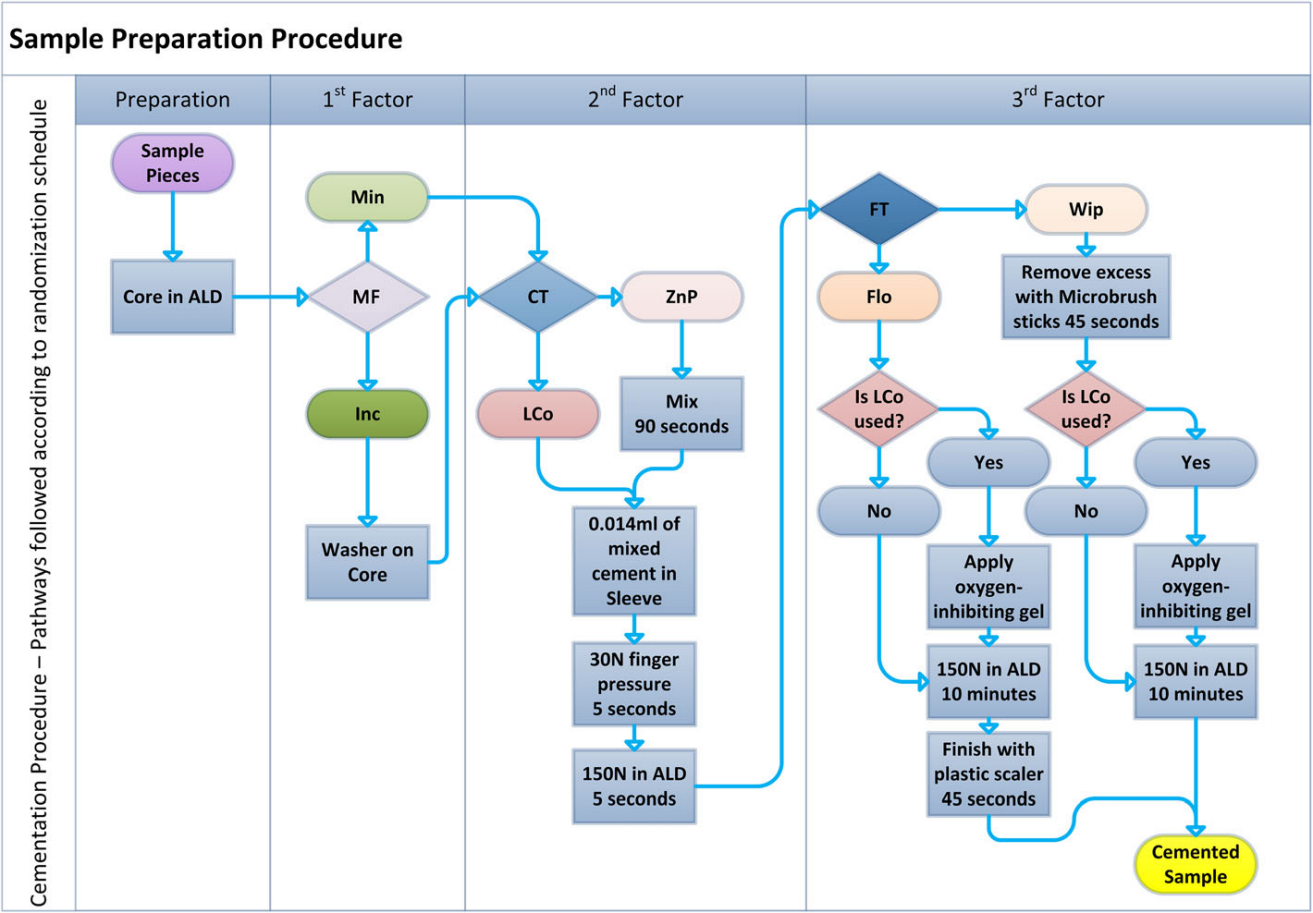
A randomly followed path that resulted in a cemented experimental crown. The abutment was secured in the Axial Loading Device (ALD) holder, a washer of known thickness was placed on the abutment to introduce a known discrepancy (Inc) or not (Min), the crown was seated on the abutment either filled with a Luting Composite (LCo) or with Zinc Phosphate (ZnP) cement, and finally a procedure was applied to remove excess cement either before setting (Wip) or after (Flo)

In a similar manner, the NSam were cemented. The following workflow, similar to the one followed for the 31 CSam, is summarized in a flowchart (Fig. 1). The first factor was the predetermined initial MF, the second factor was the cement type (CT), and the third factor was the excess cement removal technique—finishing technique (FT) (Fig. 1).

Analytically, regarding the NSam, the abutment was placed in the sample holder of the ALD. Half of the samples were cemented with their original MF (Min); the other half received a stainless steel washer to increase the MF by 75 μm (Inc). Instructions of the manufacturer were followed to mix the zinc phosphate cement (ZnP). Mixing took place on a cool and dry glass plate with a cement spatula within the indicated time frame (90 s). The luting composite (LCo) came in the QuickMix syringe, which in combination with the mixing tips, type 14, automatically mixed the material. Both materials were mixed in the same conditions (23 ± 1 °C, 50 ± 10% RH). With an insulin syringe, a standardized volume (0.014 ± 0.007 ml) of mixed luting material was applied in the crown, which then was seated on the abutment with finger force of 30 ± 3 N applied for 5 s. Then, the sample was subjected to 150 ± 0.2 N of static load in the ALD.

Excess cement was removed with the wipe-off technique (Wip), after 5 s of 150 N static load in the ALD, utilizing within 45 s two to four regular-sized microbrush sticks (Microbrush disposable micro-applicators regular size, Microbrush International, Waterford, Ireland). After cement clean-up with the wipe technique, the sample was subjected to constant load of 150 N in the ALD, for at least 10 min at room temperature (23 ± 1 °C), until the cement was completely set. The luting composite was also left to be set chemically. In order to enable its complete and homogenous curing, an oxygen-inhibiting gel (Panavia F 2.0 Oxyguard II, Kuraray Medical Inc, Okayama, Japan) was applied at the margins. Excess cement was removed with the flick-off technique (Flo) after the setting with a plastic scaler, also within 45 s.

According to this cementation protocol, seating of the crown was completed within the designated working time. Margins were always visible, and optical enhancement equipment was not used. The screw orifice at the crown component was sealed with hard wax before cementation. In this way, 120 NSam were cemented, forming 8 groups with 15 samples each: Group 1: MinZnPFlo, Group2: MinLCoFlo, Group3: MinZnPWip, Group4: MinLCoWip, Group5: IncZnPFlo, Group6: IncLCoFlo, Group7: IncZnPWip, and Group8: IncLCoWip.

### Negative replica technique

**Fig. 2:**
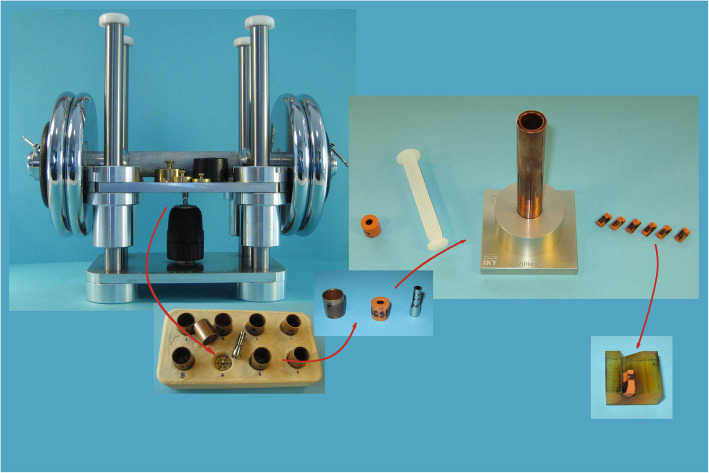
The original apparatus implemented in the study. From left to right following the arrows: A cemented experimental crown held at the Axial Loading Device (ALD), the slot at the Replica Production Device (RPD) where the cemented crown would be replicated, the acquired negative replica from within the specialized copper tray with the corresponding cemented experimental crown on the right, the negative replica inserted with a plastic plunger in the inner tube of the Sample Intersection Device (SID), and the six negative replica slices on the right, which were the observation units. Finally, each slice was placed on the Sample Observation Base (SOB) for examination under a stereomicroscope

After acquisition of the cemented sample from the ALD holder (Fig. 2), the wax, filled in the screw orifice, was removed with a sharp drill bit (Stahl HSS - G Ø2.0 mm, Craftomat, Germany). The cemented sample was placed standing fitting on a pin protruding at the center of a socket on the replica production device (RPD) (Fig. 2). A specialized copper tray was then precisely fitted in the corresponding RPD socket surrounding the cemented sample at the center. The volume between the sample and the copper tray was filled with addition silicone (Express 2- Ultra-light body quick -Vinyl Polysiloxane Impression material, 3M ESPE, Germany) with an appropriate dispenser.

The impression material was homogenously applied on the sample surface with compressed dry air. The material was left to set for at least 10 min. After setting, the tray was easily removed from its socket. The sample was removed with one move by applying pressure at the top part of the implant analog, and the NR was obtained from the tray by applying steady force in a wet environment. Finally, the acquired NR was trimmed and examined for flaws macroscopically. Next, NR was stained (Lumocolor permanent special M, Staedtler Mars GmbH & Co, Nuernberg, Germany) to enhance contrast and placed in the Sample Intersection Device (SID) for sectioning (Fig. 2).

The SID was a portable, easy to use, and robust device, manufactured by a specialized company (CNC Solutions Co., Peania, Greece). It facilitated precise sectioning of replicas in six 60° sectors in either random or oriented manner and allowed quick acquisition of sample sections afterwards. The SID was composed of two parallel concentric copper tubes fixed on an aluminum base. Six slots were opened on the long axis of the tubes forming six 60° sectors. The inner tube was rigidly fixed on the base while the outer one could rotate freely, adjusting the width of the slots. The inner diameter of the inner tube was the same with that of the specialized copper trays of the RPD.

A prepared negative replica was placed at the inner tube with a small amount of lubricant (K-Y Lubricating Jelly, Johnson & Johnson, New Brunswick, NJ, USA) and pushed at the bottom with a plastic plunger. Next, the intersection means (Double edge, platinum-chrome blades, USA) was inserted in the aligned slots above the negative replica. The slot width was adjusted to the blade width (0.1 mm) by turning the outer tube clockwise. Then, the blade was carefully pushed towards the bottom of the tubes cutting the replica in half. The slot width was increased by turning the tube counterclockwise. The blade was removed and inserted to the next slot. This procedure was repeated once more for the last pair of slots. Finally, the SID was disassembled, and the sectioned replica was removed from the inner tube with the plastic plunger. The described steps for NR production and sectioning were conducted by one researcher once.

The described workflow for the production of negative replicas and the utilized experimental equipment are illustrated (Fig. 2). The experimental crown seated under constant load in the ALD, and then a negative replica was produced in an RPD slot by filling with low viscosity addition silicone the volume between a firmly placed cemented sample and the specialized copper tray. Next, the orange cylindrical negative replica was sectioned in the SID in six sectors, each of which was placed on the Sample Observation Base (SOB) to be examined under the optical microscope (Fig. 2). Utilization of the aforementioned experimental equipment contributed substantially to the standardization of the production resulting in uniform replicas and comparable digital images. Qualitative analysis and quantitative assessment was applicable on digital images acquired during preliminary trials in a similar procedure without the aforementioned equipment, however, hindering comparisons.

### Data acquisition

Each replica section was examined under a stereomicroscope (Stemi SV11, Zeiss, Germany) with a mounted digital camera (ProgRes® C3 CCD Routine Camera, Jenoptic, Germany). Photomicrographs were captured by applying the maximum magnification available for the camera (× 390). The microscope camera was controlled by its accompanying software (ProgRES Mac CapturePro 2.7.6., Jenoptic) installed in an Apple computer (Power Mac G4, Mac OS X Version10.4.11). The illuminator (Illuminator 10 for integration in Stemi carrier equipped with a 6 V 10 W halogen bulb, Zeiss, Germany) was configured at maximum output, and additional external lighting was provided by two LED illuminators (15 W, 70 Lumen, 2700 K), minimizing exposure time and enhancing sharpness of the acquired digital images. Staining of the hollow part of the replica in previous steps also improved contrast and sharpness of the images. Also, the iris diaphragm was set open, minimizing the depth of field and increasing resolution. The resolution limit of the procedure was estimated at 0.65 μm.

Replica sections were examined on a customized epoxy resin Sample Observation Base (SOB), designed for the geometry of the observation units (replica slices) (Fig. 2). It facilitated accurate and quick placement under the stereomicroscope in a reproducible way. Captured digital images were checked for artifacts, such as bubbles and stain faults. If the outline of the sample could not be identified, previous steps were repeated. Finally, digital images were saved as .TIF files with code-name in an external USB drive. In this way, 906 digital images corresponding to 151 cemented samples were accumulated. The 151 experimental crowns were of two different types (CSam or NSam), were cemented with different luting agents, and had different initial marginal fit levels, and the excess cement was handled in different ways, in an effort to simulate the variety of settings occurring in clinical practice. Furthermore, the selected specimens presented axial symmetry, which facilitated the production of homogenous negative replicas with the aid of customized experimental equipment. The result of this homogeneity was that the 906 acquired digital images were uniform allowing standardize processing and comparisons. From those 906 images captured by one researcher, 130 were randomly selected for analysis in the present study.

### DIAS—Digital Image Analysis Sequence

**Fig. 3:**
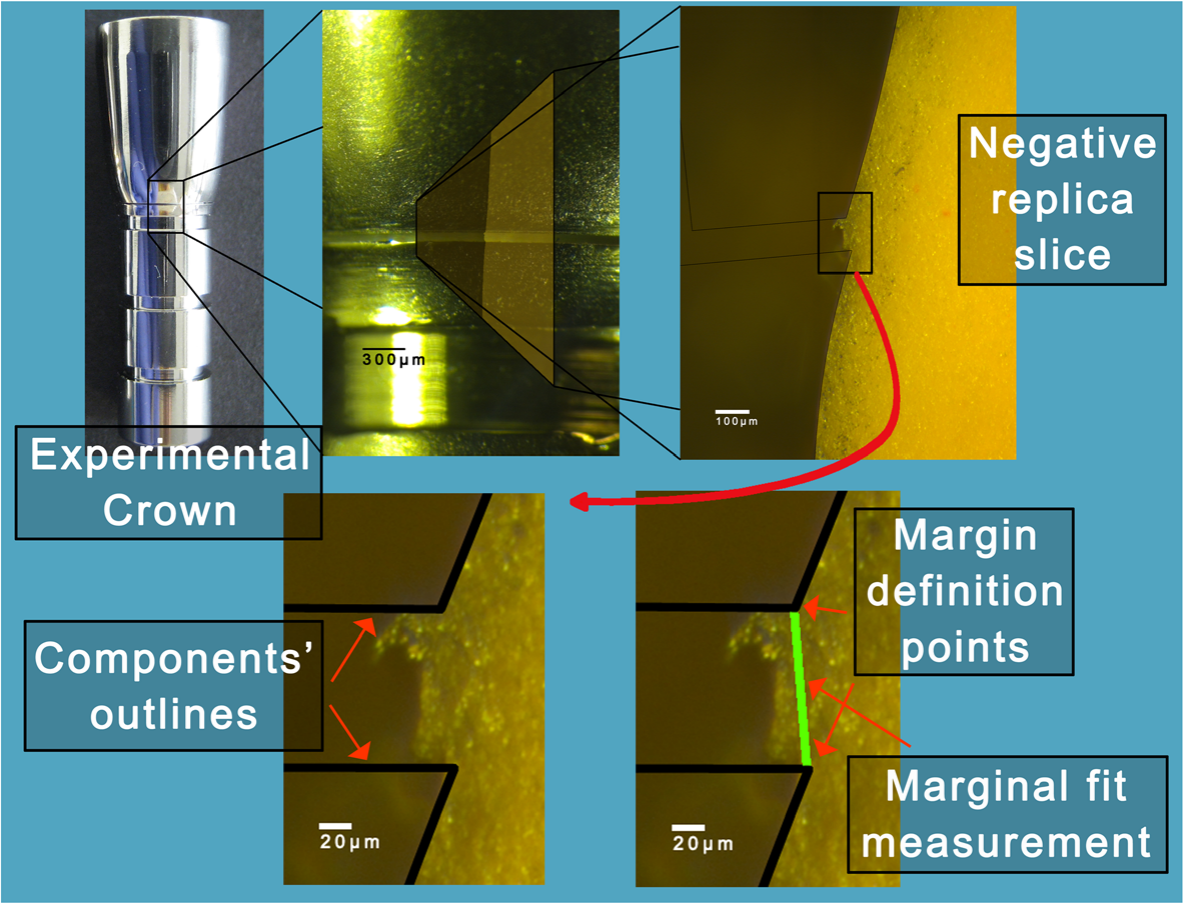
Digital Image Analysis Sequence: Images on the top raw from left to right: First, a cemented experimental crown, focus on the exposed cement surface at the margin. Second, the superimposed acquired image of the negative replica slice representing a profile parallel to the long axis of the sample and perpendicular to the margin. Next, the captured image of the negative replica slice with the superimposed components outlines (thin black lines) placed at the location of best perceived fit. Images on the bottom raw: Focus on the margin with the components’ outlines shown with thick lines. The crown outline (angled line on top) and the abutment outline (angled line at bottom). Next, the green line represents the measurement of MF accurately defined between the two angle points

Acquired digital images were analyzed in a sequence of steps. MF was defined as the line segment between two marked points on the sample profile, one located at the prosthesis margin and one at the abutment finishing line. Finally, measurements were taken. The composite Fig. 3 presents the concept of DIAS by demonstrating an experimental crown (NSam), which was cemented according to one of the pathways of the flowchart in Fig. 1. The acquired image of the negative replica slice revealed the profile of the cemented crown and enabled the orientation of the component outlines (crown-abutment), which facilitated the margin definition and marginal fit measurements (Fig. 3).

#### A. Digital tools

As an initial step for the application of DIAS, the outlines of the crown and the abutment were drawn. For each sample component, either the crown or the abutment, a NR was produced, sectioned, and photographed with the same settings under the microscope as described earlier and they were further processed with the software Adobe Photoshop (Adobe Photoshop CS 5 V12.0.4 x64, Adobe Systems Inc.). The first image was set as the background. Next, each of the following five images of the component was superimposed as a new layer with 40% transparency. Using the “Move Tool,” each superimposed image was oriented and placed to best fit the depicted component edge at the underlying image and finally the composite image was “flatten.”

The edge of the observed component at the composite image was the average profile of the component. Utilizing a highly sensitive digitizer (Bamboo CTH-470/S, Wacom, Toyonodai, Japan), a “path” was created from the tracing of the component’s average profile. Then, on the formed profile, a point was marked corresponding to the crown exposed margin or to the abutment finishing line depending on the type of the processed component outline (Fig. 3). These marks would later facilitate the MF measurements (Fig. 3). Finally, the crown outline and the abutment outline were saved as “paths” for use in the next step. This step was repeated for each of the two components of the different experimental crowns (CSam, NSam) that were used in the study and was conducted once by one researcher (observer 1). Four outlines were created in this way. The designed component outlines were utilized as a digital aid at the image processing steps that followed.

#### B. Image processing

The created component outlines were utilized to mark the profile of the cemented sample on the image of the replica slice (Fig. 3). This was possible due to the uniform geometry of the experimental crown components that were utilized in this experiment, as well as the standardized acquired digital images. First, the acquired image was open with Adobe Photoshop and a new path was created from the “paths” menu. The corresponding outline was copied from the “paths” menu of the relevant composite image of the previous step and pasted as a new path to the analyzed image. Second, the component outline (i.e., crown) was oriented and placed by the examiner at best perceived fit position on the depicted edge of the sliced replica using the “path selection tool.” Horizontal, vertical and rotation adjustments were made with the “Free Transform Path” procedure. These operations were repeated for the next component (i.e., abutment).

The next commands were “Make selection,” “Feather radius” set at zero pixels, and “Anti-aliased” option not selected. Then, from the edit menu, the “Stroke” command was selected. Line width was set at one pixel, location at the center of selection, and color black. Then, the image was saved without compression.

#### C. Measurements

The public domain Java image processing program ImageJ V1.52p (National Institutes of Health, USA) was run from its installation folder. From the main menu, the path “Edit>Options>Appearance…” and “Open images at 100%,” “Black canvas,” “IJ window always on top,” and “OK” were selected. Next, from the main menu, “Analyze>Set Measurements…” was selected. Only the option “Display label” was checked and decimal places were set at two “2” and “OK”.

From the main menu, the path “File>Open…” was selected, and after browsing, the processed images were selected and loaded in an uneven order. From the main menu, “Analyze>Set Scale…” was selected. Pixel aspect ratio was set to 1.0. The button “Click to Remove Scale” was clicked, and the box “Global” was ticked. Then, “OK.” Therefore, MF was recorded as distance in pixels between the pre-marked pixel on the crown outline and the one on the abutment outline. The outlines, which were the “digital tools” described in section A, were common for all CSam and all NSam experimental crowns on abutments and had pre-marked the margin definition points.

From the tools menu, the “Magnifying Glass” tool was selected. After a few clicks on the first image at the area where the margin definition points were marked, the image got a digital zoom up to 3200%. Pixels and the margin definition points could be clearly seen. Then the “straight line” tool was selected. The cursor was placed at the margin definition point on the crown outline, clicked and dragged towards the margin definition point on the abutment outline. Then, the measurement was automatically taken by pressing the “CTRL+M” keys and was automatically recorded in a text file. Finally, the window of the measured image was closed and the procedure was repeated for the next image. After all the measurements were taken, the results window was selected, and from the main menu, “File>Save as…” the file was saved.

DIAS was run by observer 1 twice in different sessions with a 15-day interval. Three other observers (observer 2, observer 3, and observer 4) performed the analysis independently, using the same equipment. All examiners had laboratory research experience and were previously trained for using the software Adobe Photoshop and ImageJ. However, not all of them were dentists. The 130 digital images were identified by a code and were randomly and anonymously analyzed, ensuring blinding of the examiners. Five groups with 130 measurements each were formed with this setup.

### Statistical analysis

The statistical package SPSS (SPSS for Windows, Rel. 25. 64-bit ed. 2017 Version 25. IBM Corp.) was used. Descriptive statistics were computed for the recordings of all researchers to present the distribution of the image analysis results. Normality of the data, as well as compared differences, were examined by Shapiro–Wilk and Kolmogorov–Smirnov tests, Q-Q plots, and corresponding histograms. Data were graphically represented in boxplots.

The intra- and interobserver reliability was evaluated by the ICC, examining whether the DIAS could be effectively used by a variety of different examiners. In this test, both observers and images were considered random factors. The interobserver reliability was computed by comparing the observer’s 1 mean with the results of observers 2, 3, and 4 separately. Non-parametric correlation tests Kendall’s tau (*τ*) and Spearman’s rho (*r*_s_) were also run to verify the results.

Agreement was estimated according to three parameters: The SEM, SDC_95%_, and the LoA_95%_. The restricted maximum likelihood method was implemented for the estimation of variance components necessary for the calculation of SEM. The Bland and Altman method was implemented to quantify agreement between the measurements of observer 1 with the measurements of the other three observers separately by means of a graphic representation of the calculated limits of agreement.

The type I error was set at 0.05. For each comparison, the effect sizes were also calculated. Using GPower, assuming a small effect size of *r* = 0.11, an alpha level of *α* = 0.05, and a minimum power level of *β* = 0.80, a sample size of 130 per group was a priori determined [59, 60]. Power was also calculated post hoc with the open source software for power analysis and sample size calculations GPower [59, 60].

## Results

One hundred thirty digital images were anonymously and randomly analyzed two times by observer 1 and once by each of the three other observers in order to assess the validity of the DIAS. Five groups were derived from this setup with 650 analyzed images in total. The initial MF of the samples in this study ranged between practically ideal fit (4.5 μm) and up to 205 μm. Discrepancies were artificially introduced before cementation with washers of known thickness [6]. The result of Shapiro–Wilk test of normality was *P* < .05 rejecting the null hypothesis that the data were not different from a normal distribution. The Q-Q plots, histograms, skewness, and kurtosis reflected the same result that the data are not normally distributed and exhibited positive skewness. The descriptive statistics are summarized in Table 1. The median and range are shown first in the table because the median is more representative than the mean when the data are asymmetrically distributed.

**Table 1:** Descriptive statistics of the image analysis procedure for all observers

	Observer 1—1st mes	Observer 1—2nd mes	Observer 2	Observer 3	Observer 4
Valid data	130	130	130	130	130
Shapiro–Wilk P^†^	< .001^‡a^		< .001^a^	< .001^a^	< .001^a^
Median*	132.6838	132.6550	132.1400	131.8350	132.7050
Range*	285.91	285.17	283.72	283.77	286.58
Minimum*	23.27	22.95	24.70	24.60	22.83
Maximum*	309.18	308.11	308.42	308.37	309.41
Mean*	117.1572	117.0547	117.1708	117.3008	117.1818
Std. deviation	63.99465	63.86425	63.62508	63.64398	63.99491

^*^Units are in pixels

^†^Shapiro–Wilk test of normality (all values are significant *a*)

^‡^Shapiro–Wilk test of observer 1 mean

Both the intra- and interobserver reliability had excellent ICC scores (ICC ≈ 1, Power (1-β err prob) > 99.99%) (Table 2). The applied alternative methods (non-parametric correlation tests Kendall’s tau (*τ*) and Spearman’s rho (*r*_s_)) for assessing correlation of measurements show excellent correlation as well (1). Observer 1’s mean measurements of marginal fit implementing DIAS were significantly correlated with observer 2’s measurements (*r*_s_ = .998, *τ* = .977), and observer 3’s measurements (*r*_s_ = .998, *τ* = .971), as well as observer 4’s measurements (*r*_s_ = .999, *τ* = .984) (all *P*s < .0001 and Power (1-β err prob) > 99.99%). In Table 3, the calculated SEM and SDC_95%_ values in pixels are shown.

**Table 2:** Intra- and interobserver reliability in the first step of image analysis

		Image processing^*^	
		Intraobserver ICC^†^	Interobserver ICC^†^
	ICC^‡^	1.000	1.000
95% CI	Lower bound	1.000	1.000
	Upper bound	1.000	1.000

Two-way random effects model where both people effects and measures effects are random

^*^Placement of the component outlines at the position of best fit as conceived by the observer

^†^Type A intraclass correlation coefficients using an absolute agreement definition

^‡^*ICC* intraclass correlation coefficient

**Table 3:** Intra- and interobserver agreement values

	Intraobserver	Interobserver
SEM_agreement_^†^	0.707	0.8769
SDC_95%_^‡^	1.96	2.43

^*^All units are in pixels

^†^*SEM*_*agreement*_ standard error of measurement agreement

^‡^*SDC*_*95%*_ smallest detectable change at 95% confidence interval

Tests of normality for the distribution of differences between examiners showed no significant deviations from normal distribution. The results of these tests are summarized in Table 4. Hence, parametric tests were run. The intrarater agreement was determined with the Bland and Altman analysis of bias. The Bland and Altman method included a one-sample *t* test of differences. The null hypothesis that the set of the measurements of each of the observers 2, 3, and 4 would not be different from the mean measurement of observer 1 was maintained. In the one-sample *t* test with 129 degrees of freedom, the *P* > .05 (not significant (*ns*)) indicated absence of fixed bias. The effect sizes Pearson’s *r* and Cohen’s *d*_z_ were also calculated. On average, the measurements of observer 1 in pixels (*M* = 117.11, *SE* =5.61) were not significantly different from those of observer 2 (*M* = 117.17, *SE* = 5.58, *t* = − ,687, *P* = .494 (*ns*) > .05, *r* = .06, *d*_z_ = − .06, Power (1-β err prob) = 10.47%), neither from those of observer 3 (*M* = 117.30, *SE* = 5.582, *t* = − 1.648, *P* = .102 (ns) > .05, *r* = .114, *d*_z_ = − .145, Power (1-β err prob) = 37.29%) nor from those of observer 4 (*M* = 117.18, *SE* = 5.61, *t* = − 1.109, *P* = .270(*ns*) > .05, *r* = .097, *d*_z_ = − .097, Power (1-β err prob) = 19.62%).

**Table 4:** Tests of normality—distribution of differences between examiners

	Shapiro–Wilk		
	Statistic	*df*	Sig.
Observer 1—1st	0.986	130	0,189 (*ns*)
Observer 1—2nd	0.986	130	0,189 (*ns*)
Observer 2	0.984	130	0,138 (*ns*)
Observer 3	0,995	130	0,954 (*ns*)
Observer 4	0,980	130	0,056 (*ns*)

**Fig. 4:**
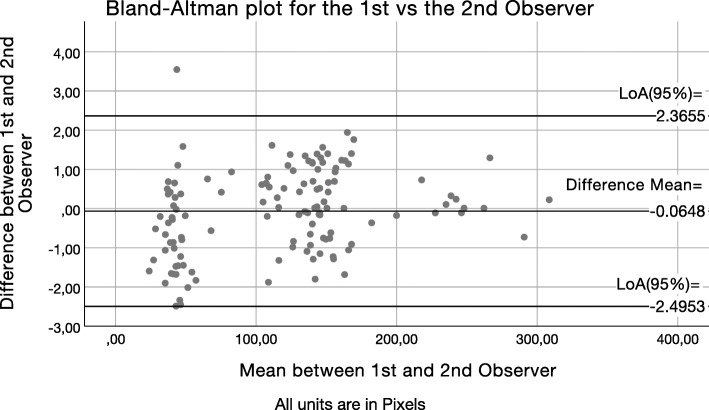
Bland–Altman scatter plot of the difference between first observer’s average measurements versus the second observer’s (A). *Y*-axis: Difference between the first observer and the second observer (A). *X*-axis: Average of their measures. Difference mean: Estimated bias between the first observer and the second observer (A). *LoA*_*95%*_ limits of agreement at the 95% confidence interval

**Fig. 5:**
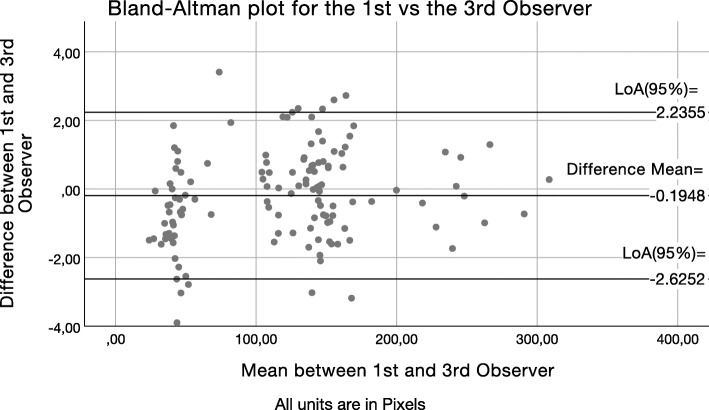
Bland–Altman scatter plot of the difference between first observer’s average measurements versus the third observer’s. *Y*-axis: Difference between the first observer and the third observer (B). *X*-axis: Average of their measures. Difference mean: Estimated bias between the first observer and the third observer (B). *LoA*_*95%*_ limits of agreement at the 95% confidence interval

**Fig. 6:**
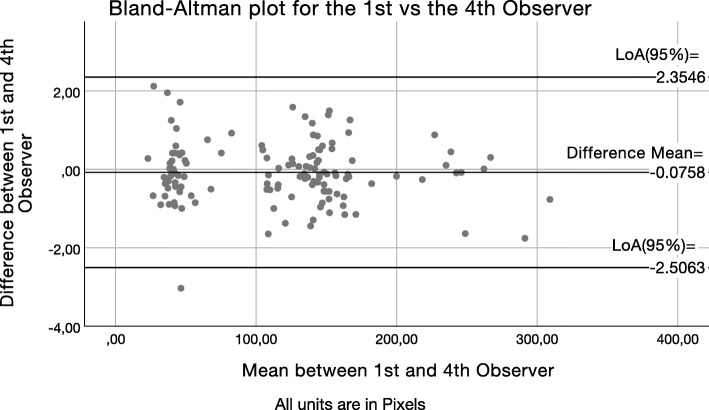
Bland–Altman scatter plot of the difference between first observer’s average measurements versus the fourth observer’s (C). *Y*-axis: Difference between the first observer and the fourth observer (C). *X*-axis: Average of their measures. Difference mean: Estimated bias between the first observer and the fourth observer (C) respectively. *LoA*_*95%*_ limits of agreement at the 95% confidence interval

After calculation of the LoA_95%_, Bland–Altman scatter plots were drawn to present graphically the level of agreement between the average measurements of observer 1 and each one of the other observers (2, 3, 4). The Bland–Altman scatter plots of the difference between the first observer’s average measurements and the second (Fig. 4), third (Fig. 5), and fourth (Fig. 6) observer’s measurements were drawn with the aid of SPSS software. The *Y*-axis illustrates the difference between the first observer’s mean and each other observer respectively (Figs. 4, 5, and 6). The *X*-axis presents the average of their measures. Difference mean is the estimated bias between the first observer’s and second (Fig. 4), third (Fig. 5), and fourth (Fig. 6) observer’s measurements, respectively. LoA_95%_ is the limits of agreement at the 95% confidence interval.

## Discussion

This study showed that the DIAS is a reliable and reproducible instrument for the quantitative evaluation of MF on cemented implant-supported crowns. In this study, the followed NR technique and the image acquisition procedure provided a high level of standardization among acquired images. Random subject variation and bias were tackled by blinding the observers with randomly assigning the images for analysis [48]. It was not possible to draw conclusions on the effects of cementation on the MF of the samples with the setup of this study.

After implementing the digital image analysis sequence (DIAS), a recently developed stepwise procedure, crown margin, and abutment finishing line as well as the profile of the set cement were recognizable on the two-dimensional image of the replica cross section [41, 42]. The marginal fit could be examined directly on cemented crowns in situ [8, 9, 34]. However, direct observation of the margin would facilitate only assessment of the vertical discrepancies and only qualitative assessment of the exposed cement surface. Examination of the margin of a cemented crown from the side is a subject of microscope’s depth of field-delicate calibration and orientation of the sample, providing images that seem to be blurry, obscuring accurate measurements. An alternative could be a destructive method with embedding the samples, cross sectioning them and observing the actual sections under the microscope [35]. However, this technique also includes many steps, is time consuming, and might also introduce artifacts at the fragile cement profile. The utilization of a scanning electron microscope would increase costs and would also be time consuming [7]. Utilization of an optical microscope and implementation of sample recycling procedures, which facilitated the reuse of specimens, in the described NR technique minimized costs. Observer subjectivity was minimized with the implementation of strict and clear criteria to identify the cement profile and the MF. Therefore, the within and between observer variation were drastically reduced.

The axial symmetry of the selected two-component samples seems to be a viable model to investigate the abutment-prosthesis system in similar studies. Additionally, the setup of this experiment facilitated standardized procedures. Furthermore, images were checked for artifacts upon acquisition, which minimized observation errors and reduced variation. Moreover, the use of a program to automatically record and organize the data eliminated the human factor from these procedures.

The authors provide a detailed description of the implemented DIAS. MF was defined as the line segment between two easily recognizable and preselected points, located at the marginal edge of each component. Furthermore, each component was accurately identified by manually placing the corresponding outline on the profile of the sectioned replica of the crown-abutment assembly. Manual image analysis procedures might be considered alternative to automated procedures. However, this step was the only source of variability, since the final step was the execution of the measurement in pixels of a well-defined line segment. Although in this study measurements were taken in pixels, ImageJ could be easily calibrated to summarize meaningful units.

In order to ensure reproducibility of results, reliability and agreement were tested with several different methods [52–54]. The Bland and Altman method facilitated the determination of the systematic error between observer 1 and each of the next three observers in this study, the investigation of the existence of any systematic difference between the measurements, and the calculation of the LoA_95%_. The estimated bias was the mean value of the examined difference. The standard deviation of the differences represented the random fluctuations around the estimated bias.

There have been studies focusing on the reliability of RT, comparing it with different methods. However, RT is different from the NR that has been used in this study, hindering comparisons. Additionally, it was not within the scope of this study to compare results deriving from different methods. Since the RT and NR methods share certain similarities such as the idea of a medium that represents the hollow space between fitted components, it is worth mentioning their findings, which are summarized in Table 5.

**Table 5:** Summary of findings of other studies

Statistics	Study	
	Rudolph et al. [36]	Boitelle et al. [31]
ICC intrarater	0.993	0.991
ICC interrater	0,978	–
Bias	− 1.3*	0.82*

*In comparison to the triple scan method

Furthermore, Mai et al. 2017 studied the agreement between the RT and a computer-aided replica technique utilizing an optical scanner [40]. The agreement estimate for the marginal fit between the two methods was 0.9999, and the precision estimate was 0.9838. Similarly, high scores of correlation statistics were found in this study; however, the context of the correlations was different, prohibiting comparisons with the aforementioned studies. In this study, differences between repeated measurements were not significant, indicating test-retest reliability and low variance of recorded scores. Also, the correlation coefficients scores, for the repeated measurements, revealed that scores between repeated measurements were strongly correlated. Nevertheless, correlation tests are not appropriate to examine agreement between observers, because they cannot reveal possible fixed bias [52].

The experimental error was exceptionally small, increasing confidence in measurements. DIAS demonstrated excellent intra- and interobserver reliability. The ICC values were ≥ 0.99, suggesting excellent reliability [46]. Similarly, high reliability scores have been observed by others that implemented image analysis in their study [44]. Some researchers argue that the SEM is a preferable measure of the quality of an assessment than is reliability, especially in studies with small sample sizes [54]. The SEM depicts the quality of an evaluation free of limitations such as sample size. The SEM both intra- and interobserver in this study was exceptionally small < 1%, which means that the presented methodology has a very low level of measurement error, increasing therefore the confidence in measurements. Otherwise stated, there is more than 95% certainty that the true value is within the ± 2% of the observed value.

In this study, the intraobserver SDC_95%_ was 1.67% and interobserver SDC_95%_ value was 2.07%. The meaning of these values is that in 95% of cases the image analysis method could detect a difference in MF if it was larger than about 2% of the mean MF of the total sample. The minor differences between observer 1 and the other three observers that took part in this study might be attributed to the familiarization with the method. Observer 1 had greater experience with the method in comparison with the other three observers despite the fact that they had received adequate training and conducted several trials before they run the analysis. An additional reason might be the motivation to take accurate measurements, because the suggested method is time consuming, requires commitment and precision.

Due to the very low probability that different observers would perfectly agree and proceed with identical ratings for a certain set of measurements, it was examined if different observers would give significantly different ratings with the DIAS. The Bland and Altman method was applied to estimate the likelihood of each observer to give a different measurement from those of observer 1. This approach is often used to examine the limits of agreement between two different methods or different examiners [52, 53]. In order to calculate with a certain level of precision the limits of agreement between two examiners, a relatively large number of observations is required. The sample size in this study was *N* = 130, which was adequate and reasonably large according to Bland’s recommendations.

The bias in repeated measurements between examiners did not exceed 0.2% (0.166%), and the paired-samples *t* test could not reveal any bias (*ns*) at the studied sample size, although the inability to show that a difference exists at the acquired data is not a proof of absence of such difference. Furthermore, the calculated power of the *t* tests was small (10.47–37.29%); however, the effect sizes (*r* and *d*_z_) were also small [61, 62]. A small effect size indicates that if a difference actually exists, it might be revealed in a very large sample size and it would be so small that it would not have any practical meaning eventually. The GPower software was utilized for the post hoc calculation of sample size and power [56, 57]. The calculated sample size for parametric tests with adequate power for such effect sizes was more than 2000 samples.

Observers 2, 3, and 4 had, respectively, one, zero, and four outliers in their measurement differences from observers 1’s averaged measurements. While observers 2 and 4 had outliers in their measurement differences, they also had a better agreement with observers 1’s measurements than what observer 3 did. Additionally, the three Bland and Altman scatter plots show very good agreement with minimal observations laying outside the LoA boundaries. Even differences within the ± 5 pixels range, which is larger than the observed accuracy in this study, would not have any practical significance. When transforming the observed discrepancies in meaningful units (0.65 μm/pixel) at the calibrated images of this study, the ± 5pixels range would represent a maximum discrepancy of about 6 μm, which does not seem to have practical value clinically [11].

## Limitations

This was a laboratory study, conducted in relatively stable environmental conditions, which is not the case when cementation takes place in vivo. The applied DIAS was based on identical and of favorable shape (axial symmetry) implant-components. Therefore, the method might be problematic for fixed prostheses on natural teeth. The analysis was based on two-dimensional observations, posing an additional limitation. The examined DIAS was time consuming and required familiarization of the researcher [37].

Further large-sized clinical trials are needed to verify the results and to evaluate feasibility of application in clinical conditions. Combination of the method with pattern recognition software could reduce the required time for an accurate placement of the outlines on the depicted cemented sample profile.

## Clinical relevance

Since the NR technique is non-destructive, it has the potential to be implemented in both ex vivo and in vivo studies. The NR technique in combination with DIAS has the advantage to provide a precision representation of the prosthesis-abutment assembly, as well as their exposed interface, contributing to the quality control of various steps of prosthesis manufacturing. The achieved marginal fit is one magnitude that can be measured with the combination of these reliable techniques. However, accessing the margin in a manner to partially include the surface of the abutment without traumatizing the delicate peri-implant tissues requires a reproducible method that is yet to be verified clinically.

In clinical situation, a NR might be an impression extending to the abutment surface. Due to the dimension stability of modern impression materials, the acquired replica could be sectioned and studied post hoc without burdening the workflow of a busy practice. Furthermore, image acquisition is a technical procedure which does not require medical personnel. Additionally, the DIAS is also possible to be carried out by a non-medical personnel as did in this study. The combination of NR and DIAS can be a valuable tool to evaluate the achieved marginal fit of cemented crowns and assess the quality of cementation, contributing to the quality improvement of delivered prostheses.

## Conclusions

Within the limitations of this study, the presented DIAS has excellent intra- and interobserver reliability, small measurement error and sensitivity level that covers the relevant research needs. Additionally, with the prerequisite of axial symmetry of analyzed components, the method could be applied to at least two designs. Furthermore, the negative replica technique is a highly standardized and cost-effective approach for the evaluation of marginal fit. The DIAS in combination with the NR technique is an objective and reliable method with high level of standardization, able to detect and quantify MF observed in clinical practice.

## Data Availability

The dataset used and analyzed during the current study are available from the corresponding author on reasonable request (DIAS Validation raw DATA 4 IJID.sav)

## Abbreviations

ALD: Axial loading device
CSam: Two-component abutment Friadent CeraBase D5.5mm
DIAS: Digital Image Analysis Sequence
Flo: Flick-off technique
ICC: Intraclass correlation coefficient
Inc: Increased initial marginal fit
LCo: Self-adhesive dual-curing luting composite
LED: Light-emitting diode
LoA: Bland and Altman method of limits of agreement
MF: Marginal fit
Min: Original marginal fit
NR: Negative replica
ns: Not significant
NSam: Simplified sample Novadental
RH: Relative humidity
RPD: Replica production device
RT: Replica technique
SDC_95%_: Smallest detectable change at the 95% confidence level
SEM: Standard error of measurement
SID: Sample Intersection Device
SOB: Sample Observation Base
SPSS: Statistical Package for the Social Sciences
TIF: Tagged Image File
USB: Universal Serial Bus
Wip: Wipe-off technique
ZnP: Zinc phosphate cement

## References

[1] Teichmann M, Göckler F, Rückbeil M, Weber V, Edelhoff D, Wolfart S (2019). Periodontal outcome and additional clinical quality criteria of lithium-disilicate restorations (Empress 2) after 14 years. Clin Oral Investig.

[2] Laurent M, Scheer P, Dejou J, Laborde G (2008). Clinical evaluation of the marginal fit of cast crowns--validation of the silicone replica method. J Oral Rehabil.

[3] Holmes JR, Bayne SC, Holland GA, Sulik WD (1989). Considerations in measurement of marginal fit. J Prosthet Dent.

[4] Pimenta MA, Frasca LC, Lopes R, Rivaldo E (2015). Evaluation of marginal and internal fit of ceramic and metallic crown copings using x-ray microtomography (micro-CT) technology. J Prosthet Dent.

[5] Wadhwani C, Goodwin S, Chung KH (2016). Cementing an implant crown: a novel measurement system using computational fluid dynamics approach. Clin Implant Dent Relat Res.

[6] Jacobs MS, Windeler AS (1991). An investigation of dental luting cement solubility as a function of the marginal gap. J Prosthet Dent.

[7] Oyagüe RC, Sánchez-Turrión A, López-Lozano JF, Suárez-García MJ (2012). Vertical discrepancy and microleakage of laser-sintered and vacuum-cast implant-supported structures luted with different cement types. J Dent.

[8] Gonzalo E, Suárez MJ, Serrano B, Lozano JF (2009). A comparison of the marginal vertical discrepancies of zirconium and metal ceramic posterior fixed dental prostheses before and after cementation. J Prosthet Dent.

[9] Linkevicius T, Vindasiute E, Puisys A, Peciuliene V (2011). The influence of margin location on the amount of undetected cement excess after delivery of cement-retained implant restorations. Clin Oral Implants Res.

[10] Kim EH, Lee DH, Kwon SM, Kwon TY (2017). A Microcomputed tomography evaluation of the marginal fit of cobalt-chromium alloy copings fabricated by new manufacturing techniques and alloy systems. J Prosthet Dent.

[11] Tosches NA, Brägger U, Lang NP (2009). Marginal fit of cemented and screw-retained crowns incorporated on the Straumann (ITI) Dental Implant System: an in vitro study. Clin Oral Implants Res.

[12] Bronson MR, Lindquist TJ, Dawson DV (2005). Clinical acceptability of crown margins versus marginal gaps as determined by pre-doctoral students and prosthodontists. J Prosthodont.

[13] Oyagüe RC, Turrión AS, Toledano M, Monticelli F, Osorio R (2009). In vitro vertical misfit evaluation of cast frameworks for cement-retained implant-supported partial prostheses. J Dent.

[14] Tabesh M, Nejatidanesh F, Savabi G, Davoudi A, Savabi O, Mirmohammadi H (2020). Marginal adaptation of zirconia complete-coverage fixed dental restorations made from digital scans or conventional impressions: a systematic review and meta-analysis. J Prosthet Dent.

[15] Mello CC, Lemos CAA, de Luna Gomes JM, Verri FR, Pellizzer EPCAD (2019). CAM vs conventional technique for fabrication of implant-supported frameworks: a systematic review and meta-analysis of in vitro studies. Int J Prosthodont.

[16] Sadid-Zadeh R, Katsavochristou A, Squires T, Simon M (2020). Accuracy of marginal fit and axial wall contour for lithium disilicate crowns fabricated using three digital workflows. J Prosthet Dent.

[17] Keith SE, Miller BH, Woody RD, Higginbottom FL (1999). Marginal discrepancy of screw-retained and cemented metal-ceramic crowns on implants abutments. Int J Oral Maxillofac Implants.

[18] Heckmann SM, Karl M, Wichmann MG, Winter W, Graef F, Taylor TD (2004). Cement fixation and screw retention: parameters of passive fit. An in vitro study of three-unit implant-supported fixed partial dentures. Clin Oral Implants Res.

[19] Board of Trustees of the American Academy of Periodontology (2013). Peri-implant mucositis and peri-implantitis: a current understanding of their diagnoses and clinical implications. J Periodontol.

[20] Korsch M, Marten SM, Walther W, Vital M, Pieper DH, Dötsch A (2018). Impact of dental cement on the peri-implant biofilm-microbial comparison of two different cements in an in vivo observational study. Clin Implant Dent Relat Res.

[21] Gehrke P, Bleuel K, Fischer C, Sader R (2019). Influence of margin location and luting material on the amount of undetected cement excess on CAD/CAM implant abutments and cement-retained zirconia crowns: an in-vitro study. BMC Oral Health.

[22] García-Minguillán G, Del Río J, Preciado A, D Lynch C, Castillo-Oyagüe R (2020). Impact of the retention system of implant fixed dental restorations on the peri-implant health, state of the prosthesis, and patients' oral health-related quality of life. J Dent.

[23] Hermann JS, Buser D, Schenk RK, Schoolfield JD, Cochran DL (2001). Biologic width around one- and two-piece titanium implants. Clin Oral Implants Res.

[24] Tan K, Pjetursson BE, Lang NP, Chan ES (2004). A systematic review of the survival and complication rates of fixed partial dentures (FPDs) after an observation period of at least 5 years. Clin Oral Implants Res.

[25] Reich S, Uhlen S, Gozdowski S, Lohbauer U (2011). Measurement of cement thickness under lithium disilicate crowns using an impression material technique. Clin Oral Investig.

[26] Alqutaibi AY (2017). Cement- and screw-retained implant-supported restorations showed comparable marginal bone loss and implant survival rate. J Evid Based Dent Pract.

[27] Weigl P, Saarepera K, Hinrikus K, Wu Y, Trimpou G, Lorenz J (2019). Screw-retained monolithic zirconia vs. cemented porcelain-fused-to-metal implant crowns: a prospective randomized clinical trial in split-mouth design. Clin Oral Investig.

[28] Taylor TD, Agar JR (2002). Twenty years of progress in implant prosthodontics. J Prosthet Dent.

[29] Gómez-Polo M, Ortega R, Gómez-Polo C, Celemin A, Del Rio Highsmith J (2018). Factors affecting the decision to use cemented or screw-retained fixed implant-supported prostheses: a critical review. Int J Prosthodont.

[30] Hamed MT, Abdullah Mously H, Khalid Alamoudi S, Hossam Hashem AB, Hussein NG (2020). A systematic review of screw versus cement-retained fixed implant supported reconstructions. Clin Cosmet Investig Dent.

[31] Boitelle P, Tapie L, Mawussi B, Fromentin O (2018). Evaluation of the marginal fit of CAD-CAM zirconia copings: comparison of 2D and 3D measurement methods. J Prosthet Dent.

[32] Boitelle P, Mawussi B, Tapie L, Fromentin O (2014). A systematic review of CAD/CAM fit restoration evaluations. J Oral Rehabil.

[33] Nawafleh NA, Mack F, Evans J, Mackay J, Hatamleh MM (2013). Accuracy and reliability of methods to measure marginal adaptation of crowns and FDPs: a literature review. J Prosthodont.

[34] Gassino G, Barone Monfrin S, Scanu M, Spina G, Preti G (2004). Marginal adaptation of fixed prosthodontics: a new in vitro 360-degree external examination procedure. Int J Prosthodont.

[35] Son K, Lee S, Kang SH, Park J, Lee KB, Jeon M, Yun BJ (2019). A comparison study of marginal and internal fit assessment methods for fixed dental prostheses. J Clin Med.

[36] Rudolph H, Ostertag S, Ostertag M, Walter MH, Luthardt RG, Kuhn K (2018). Reliability of light microscopy and a computer-assisted replica measurement technique for evaluating the fit of dental copings. J Appl Oral Sci.

[37] Att W, Komine F, Gerds T, Strub JR (2009). Marginal adaptation of three different zirconium dioxide three-unit fixed dental prostheses. J Prosthet Dent.

[38] Wolfart S, Wegner SM, Halabi AA, Kern M (2003). Clinical evaluation of marginal fit of a new experimental all-ceramic system before and after cementation. Int J Prosthodont.

[39] Coli P, Karlsson S (2004). Fit of a new pressure-sintered zirconium dioxide coping. Int J Prosthodont.

[40] Mai HM, Lee KE, Ha JH, Lee DH (2018). Effects of image and education on the precision of the measurement method for evaluating prosthesis misfit. J Prosthet Dent.

[41] Villias A, Niedermeier W (2014). Finishing effects on cement surfaces at different marginal fit levels. [abstract]. J Dent Res.

[42] Villias A, Niedermeier W (2014). Influence of three factors on cement profile. [abstract]. J Dent Res.

[43] Colpani JT, Borba M, Bona AD (2013). Evaluation of marginal and internal fit of ceramic crown copings. Dent Mater.

[44] Rahme HY, Tehini GE, Adib SM, Ardo AS, Rifai KT (2008). In vitro evaluation of the "replica technique" in the measurement of the fit of Procera crowns. J Contemp Dent Pract.

[45] Segerström S, Wiking-Lima de Faria J, Braian M, Ameri A, Ahlgren C (2019). A validation study of the impression replica technique. J Prosthodont.

[46] Falk A, Vult von Steyern P, Fransson H, Thorén MM (2015). Reliability of the impression replica technique. Int J Prosthodont.

[47] Mai HN, Lee KE, Lee KB, Jeong SM, Lee SJ, Lee CH, An SY, Lee DH (2017). Verification of a computer-aided replica technique for evaluating prosthesis adaptation using statistical agreement analysis. J Adv Prosthodont.

[48] Rose G, Barker DJ (1978). Epidemiology for the uninitiated. Observer variation. Br Med J.

[49] Koo TK, Li MY (2016). A guideline of selecting and reporting intraclass correlation coefficients for reliability research. J Chiropr Med.

[50] Lin L, Hedayat AS, Sinha B, Yang M (2002). Statistical methods in assessing agreement. Models, Issues, and Tools. J Am Stat Assoc.

[51] Shrout PE, Fleiss JL (1979). Intraclass correlations: uses in assessing rater reliability. Psychol Bull.

[52] Field A (2009). Discovering Statistics Using SPSS.

[53] Whitley E, Ball J (2002). Statistics review 1: presenting and summarising data. Crit Care.

[54] Bland JM, Altman DG (1999). Measuring agreement in method comparison studies. Stat Methods Med Res.

[55] Bland JM, Altman DG (1986). Statistical methods for assessing agreement between two methods of clinical measurement. Lancet..

[56] de Vet HC, Terwee CB, Knol DL, Bouter LM (2006). When to use agreement versus reliability measures. J Clin Epidemiol.

[57] Tighe J, McManus IC, Dewhurst NG, Chis L, Mucklow J (2010). The standard error of measurement is a more appropriate measure of quality for postgraduate medical assessments than is reliability: an analysis of MRCP(UK) examinations. BMC Med Educ.

[58] Nakagawa S, Johnson PCD, Schielzeth H (2017). The coefficient of determination R2 and intra-class correlation coefficient from generalized linear mixed-effects models revisited and expanded. J R Soc Interface.

[59] Faul F, Erdfelder E, Lang AG, Buchner A (2007). G*Power 3: a flexible statistical power analysis program for the social, behavioral, and biomedical sciences. Behav Res Methods.

[60] Faul F, Erdfelder E, Buchner A, Lang AG (2009). Statistical power analyses using G*Power 3.1: tests for correlation and regression analyses. Behav Res Methods.

[61] Sullivan GM, Feinn R (2012). Using Effect Size-or Why the P Value Is Not Enough. J Grad Med Educ.

[62] Cohen J (1992). A power primer. Psychol Bull.

